# Cancer cachexia: an orphan with a future

**DOI:** 10.1002/jcsm.12401

**Published:** 2019-03-28

**Authors:** Mitja Lainscak, Giuseppe M. C. Rosano

**Affiliations:** ^1^ Division of Cardiology General Hospital Murska Sobota Murska Sobota Slovenia; ^2^ Faculty of Medicine University of Ljubljana Ljubljana Slovenia; ^3^ IRCCS San Raffaele Roma St George's Hospital Medical School, University of London UK

**Keywords:** Cancer cachexia, Prevalence, Orphan Disease, Regulatory Pathway

Malignant diseases, as all chronic diseases, follow a distinct and specific trajectory that can lead to development of cachexia. When cachexia develops, patients will often have a stage of the disease with limited life expectancy. There are no proven and clinically effective interventions to improve quality of life and/or life expectancy in patients with cachexia.^1^ Despite implications for daily practice, the epidemiology data remain scarce, and several features still are largely estimates rather than robust information retrieved from cross‐sectional or prospective observational studies.^2^

In this context, contribution by Anker *et al*. in this issue of the journal^3^ is extremely timely and instructive, thus representing a big important piece in the cancer cachexia jigsaw. It is the first systematic review about cancer cachexia; authors included 21 studies with >31 000 patients, and cancer cachexia prevalence ranged from 11% to 74%. This information was then used for estimation of cancer cachexia absolute number in the USA and European Union (EU), respectively. Overall, a total of 800 300 patients (15.8 subjects per 10 000 people of the total EU population) and 527 100 patients (16.5 per 10 000 people of the total US population) are estimated to suffer from cancer cachexia. Although based on some assumptions (that usually are needed in diseases defined by arbitrary decisions), this is the first systematic review about cancer cachexia burden. Findings can be challenged because various cachexia definitions were applied, but studies were, nevertheless, conducted by those with specific interest in the issue and can be regarded as representative enough. Also, cachexia definition changed over time,^2^, ^4^, ^5^ and no good comparative studies of various cachexia definitions are available. An important observation is the fact that in absolute terms, cachexia of individual malignancy qualifies as orphan disease, with the prevalence range from 0.3/10 000 to 3.0/10 000 people of the total population. Although the orphan disease definition should not be used as a Trojan horse to bring into the market drugs at higher prices and to later expand their indication into other therapeutic areas, the identification of cancer cachexia as an orphan disease is novel and can change the landscape of drug development in this area.

One of key messages from the paper by Anker *et al*.^3^ to be picked up by the research community and clinicians should be that we need better epidemiological data. As suggested previously, all available resources should be exploited for use, including big data approach, and experience from other disease should be considered as well.^6^, ^7^, ^8^, ^9^ With existing International Classification of Disease code, only few studies exist.^10^ Nonetheless, because several countries have such nationwide resources,^11^, ^12^, ^13^ these could be source of valuable information. Some ongoing projects linking existing national databases to get insight into the current clinical practice and physician attitudes towards cachexia will provide valuable information.^14^ Another pathway to be explored is vigilant application of adopted cachexia definition in cross‐sectional, observational, and randomized trials involving cancer patients. When combined with outcome data (*Figure* *1*), this would greatly increase our understanding of epidemiological burden and prognostic implications of this grim condition.

**Figure 1:**
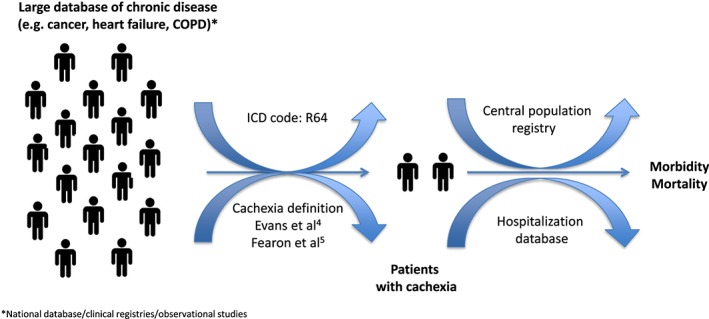
Prevalence and outcome of cardiac cachexia.

Although the work by Anker *et al*.^3^ is focusing on the epidemiology and prevalence in particular, the implications should be viewed in a much broader perspective. We definitely should argue that reporting cachexia (of chronic disease) is not a part of daily routine. This can be due to lack of awareness or, even worse, knowledge about cachexia. Also, it can be due to lack of any financial implications linked with the coding of cachexia. Can this be overcome? We likely have a strong case for cancer cachexia, but more effort should be given to increase awareness and to promote diagnostic criteria that can be applied in daily practice.^4^, ^5^ However, cancer cachexia is only one aspect of the syndrome, as cachexia in other clinical conditions as heart failure and chronic obstructive pulmonary disease can be deadlier than cancer cachexia.

In line with cancer but also other disease awareness campaigns,^15^ a worldwide action to highlight cachexia or more generally the body wasting could be initiated. The effects could then be followed with regular analysis of existing databases and scientific publications describing cachexia. Importantly, similar strategies should be implied in the case of sarcopenia, which just recently received the ICD code^16^ and has several implications that are similar to those of cachexia. Simultaneous step would be to promote (epidemiological) studies in cancer cachexia, with particular emphasis on pathophysiology and patient‐related outcomes. At the moment, we do not have any remedy to make patients with cancer cachexia better. Yet there are some encouraging reports^17^, ^18^ that could influence future efforts. Combined with the current review,^3^ maybe the most important learning is that we need to consider cancer cachexia as an orphan disease. As a result, drug development pathways may be simplified with possibility of earlier access programmes.^19^ Both European Medicines Agency and Food and Drug Administration have specific procedures in place that facilitate timely and more cost‐effective translation of basic science into clinical research and bedside use (*Figure* *2*). The European Medicines Agency has recently launched the Priority Medicines (PRIME) scheme 20 to foster research on and development of medicines for patients whose diseases cannot be treated or who need better treatment options. The PRIME scheme aim is to support and optimize drug development in order to provide a faster approval process for those molecules that show reasonable efficacy and safety profiles. However, the main problem with early access programmes is that often adequate pricing can be difficult to agree because of the greed of companies and unwillingness of the health systems to pay too much for uncertain effectiveness. In order to overcome these problems, the PRIME scheme includes the possibility for the companies to receive scientific advices together with early health technology assessments and opinion from patient groups. This new regulatory pathway has already had and will have in the future favourable effects on drug development and with meaningful clinical implications for the therapeutic areas significantly affected by cachexia.

**Figure 2:**
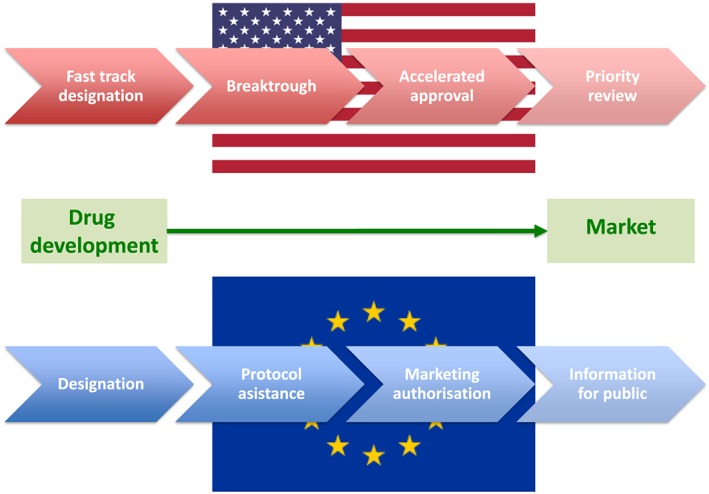
Regulatory pathways for orphan disease drug development in European Union and in the USA.

## Funding

The authors acknowledge the project (Burden of cachexia and sarcopenia in patients with chronic diseases: epidemiology, pathophysiology and outcomes; ID J3‐9292) was financially supported by the Slovenian Research Agency.

## Conflict of interest

ML reports owning shares of Actimed Therapeutics.
