# Corrigendum

**DOI:** 10.1002/jcsm.12605

**Published:** 2020-08-18

In [1], the affiliation of the author Giuseppe M. C. Rosano is incorrect. The Author and affiliation should read as follows:

Giuseppe M. C. Rosano^3^.

^3^Centre for Clinical and Basic Research, Department of Medical Sciences, IRCCS San Raffaele Pisana, Rome, Italy.
